# Effects of virtual reality-based cue exposure therapy on craving and physiological responses in alcohol-dependent patients-a randomised controlled trial

**DOI:** 10.1186/s12888-023-05426-z

**Published:** 2023-12-18

**Authors:** Junjun Zhang, Ming Chen, Junli Yan, Chaojun Wang, Hongdu Deng, Jiali Wang, Jiapeng Gu, Dan Wang, Wenhui Li, Chuansheng Wang

**Affiliations:** 1 Department of Psychiatry, The Second Affiliated Hospital of Xinxiang Medical University, 207# QianJin Road, Xinxiang, Henan 453000 China; 2 Department of Community Health, Advanced Medical and Dental Institute, Universiti Sains Malaysia, Kepala Batas, Pulau Pinang Malaysia

**Keywords:** Virtual reality, Cue exposure treatment, Craving, Physiological responses, Alcohol dependence

## Abstract

**Background:**

Cue exposure therapy is used to treat alcohol dependence. However, its effectiveness is controversial due to the limitations of the clinical treatment setting. Virtual reality technology may improve the therapeutic effect. The aim of this study is to explore whether virtual reality-based cue exposure therapy can reduce the psychological craving and physiological responses of patients with alcohol dependence.

**Methods:**

Forty-four male alcohol-dependent patients were recruited and divided into the study group (n = 23) and the control group (n = 21) according to a random number table. The control group received only conventional clinical treatment for alcohol dependence. The study group received conventional clinical treatment with the addition of VR cue exposure (treatment). The primary outcome was to assess psychological craving and physiological responses to cues of patients before and after treatment.

**Results:**

After virtual reality-based cue exposure therapy, the changes in VAS and heart rate before and after cue exposure in the study group were significantly lower than those in the control group (*P* < 0.05), while the changes in skin conductance and respiration between the study group and the control group were not significantly different (*P* > 0.05). The changes in VAS and heart rate before and after cue exposure in the study group were significantly lower than those before treatment (*P* < 0.05), while the changes in skin conductance and respiration were not significantly different from those before treatment (*P* > 0.05). The changes in VAS, heart rate, skin conductance and respiration before and after cue exposure in the control group were not significantly different from those before treatment (*P* > 0.05).

**Conclusion:**

Virtual reality-based cue exposure therapy can reduce the psychological craving and part of the physiological responses of alcohol-dependent patients during cue exposure in the short term and may be helpful in the treatment of alcohol dependence.

**Trial registration:**

The study protocol was registered at the China Clinical Trial Registry on 26/02/2021 (www.chictr.org.cn; ChiCTR ID: ChiCTR2100043680).

## Introduction

Alcohol dependence (AD) is a complex chronic relapsing disorder, which refers to a special psychological state of craving for alcohol caused by long-term, repeated drinking, as well as a special psychological and physical responses to reducing or stopping drinking [1]. Craving for alcohol is one of the core features of AD, and plays an important role in the formation, development, maintenance and relapse of AD [2–5]. However, the etiology of craving is not fully understood. From a psychological perspective, craving is acquired through repeated behaviours and positive emotional valence [6]. When the unconditioned stimulus is repeatedly associated with neutral stimulus (such as bottles of wine) and accompanied by positive reinforcement, the neutral stimulus itself will produce a response that is similar to the unconditioned stimulus, at which point the neutral stimulus is referred to as the conditioned stimulus [7, 8]. Once this reflex has been established, AD patients would experience craving when exposed to alcohol-related cues.

### Cue reactivity

Numerous studies have demonstrated that AD patients exhibit a variety of psychological and physiological responses when exposed to alcohol-related cues, such as alcoholic beverages, alcohol-related environment, emotional factors, and social interactions, collectively referred to as cue reactivity. The observed changes include changes in subjective craving [9, 10], mood [11], eye movements [12], and changes in autonomic nervous system activity, particularly heart rate, skin conductance, respiration, salivation, and blood pressure [13–19]. Furthermore, physiological responses to alcohol-related cues may be considered objective markers of cue reactivity. However, most previous studies on alcohol-related cues have focused on subjective craving and emotions, with fewer studies on physiological responses.

### Cue exposure therapy

Cue exposure therapy (CET) is a treatment method based on Pavlov’s theory of conditioning, in which AD patients are repeatedly exposed to alcohol-related cues and environments without exposure to alcohol, in order to reduce or even eliminate the conditioning between alcohol-related cues and psychophysiological responses, and to reduce the likelihood of relapse [20]. However, there is controversy about the therapeutic efficacy of CET for AD due to the fact that most treatments in clinical settings are delivered by presenting only one or two cues at a time in a controlled environment, whereas the associated cues that induce craving are varied, which apparently interferes with the therapeutic efficacy [21, 22].

### Virtual reality-based cue exposure therapy

Virtual reality (VR) is a practical technology that combines virtualisation and reality to create a digital environment that is similar to the real environment [23]. Over the past 20 years, VR has been widely used in the study of a variety of mental disorders, such as anxiety disorders [24] (especially phobias) [25, 26], post-traumatic stress disorder [27, 28], substance use disorders [29, 30], gambling disorders [31, 32], eating disorders [33, 34], and autism spectrum disorders [35, 36]. Virtual reality-based cue exposure therapy (VR-CET) has significant advantages, as VR can not only create more realistic life scene through multiple sensory inputs such as vision, auditory, smell, or touch, but also provide multiple cues for exposure simultaneously, increasing the the realism and immersion of exposure. It has been successfully used as treatment tools for anxiety disorders [37, 38], post-traumatic stress disorder [39], addictive disorders [40, 41], eating disorders [33, 34].

Although some studies have shown that VR-CET can reduce craving in AD patients, few studies have evaluated the changes in physiological responses in AD patients. There is still a lack of clear answers to the intervention effectiveness of VR-CET for AD patients. The purpose of this study is to explore the effects of VR-CET on the subjective craving and physiological responses of AD patients, and to objectively evaluate the effectiveness of VR-CET. We hypothesise that VR-CET has the potential to reduce psychological craving and physiological responses during cue exposure in AD patients and may contribute to the treatment of AD.

## Methods

### Preliminary survey and construction of cues and scene

#### Preliminary survey of cues and scene

Due to the differences in drinking culture in different countries, it is first and foremost important to identify alcohol-related cues that can effectively induce the alcohol craving in Chinese AD patients and to construct the VR cue exposure scene that is suitable for the actual situation of Chinese AD patients.

A semistructured interview was conducted with male AD patients who were hospitalized in the Fifth Department of Psychiatry, Second Affiliated Hospital of Xinxiang Medical University from September 2021 to November 2021. The interview content mainly included the basic information and alcohol consumption of the patients, including the type of alcoholic beverages, the time of drinking, the reason for drinking, the place of drinking, and whether others were present.

A total of 40 male AD patients were recruited, and all of them met the diagnostic criteria for AD in the International Classification of Diseases, 10th edition (ICD-10). The age distribution ranged from 26 to 52 years old, years of education ranged from 6 to 18 years, cumulative drinking time ranged from 3 to 28 years, addiction time ranged from 0.25 to 17 years, and alcohol intake ranged from 76 to 636 g/day.

The results showed that AD patients preferred to drink at home (37/40) and in restaurants (33/40), preferred white wine (39/40) and beer (35/40), and preferred to drink alcohol in the evening (38/40). More than half of AD patients (25/40) said that “when eating with friends in a restaurant, especially when they were persuaded to drink, they would have strong alcohol craving”. Nearly half of AD patients (19/40) said that they wanted to drink more when they saw bottles, glasses, smelled alcohol or saw others drinking, especially when they were in a restaurant.

Based on these results, we chose the setting of multiple friends having dinner and drinking in a private room in a restaurant as a cue exposure scene.

#### Construction of the VR cue exposure scene

##### Actor selection

Although the drinking patterns of social drinkers differ from those of AD patients, most AD patients develop from social drinking. We invited 5 social drinkers to participate in the shooting of the VR cue exposure **scene**, 3 of whom were healthcare workers who had already worked in the Centre for Addiction Medicine for more than 5 years and had a certain understanding of the clinical manifestations and psychological activities of AD patients, to ensure the authenticity of the **scene** as much as possible.

##### Site selection

We chose to shoot in a private room in a Chinese restaurant. The specific environmental requirements of the venue are as follows: ① the area of the private room is close to 15 square metres (4 m × 4 m); ② the round turntable table and chairs in the private room are placed in an orderly manner; ③ the lighting in the private room is bright, soft and moderate, and cannot be too bright or too dark.

##### Plot design

The plot is designed for 5 friends who have reunited after a long absence, having dinner in a private room in a restaurant. 5 people are seated around a circular table, upon which there are glasses, cutlery, white wine, beer, food and so on. In the course of the dinner, alcohol was persuaded for various reasons, such as special occasions, years of relationship, and drinking to reduce stress. Friends pour drinks, toast and discuss the taste of the wine.

##### Instrument

The Insta360Pro2 panoramic camera from Insta360 Innovation Technology Co., Ltd. was used to shoot the VR cue exposure scene, and the video was processed and imported into the Oculus quest VR equipment manufactured by Oculus for playback.

#### Validity of the VR cue exposure scene

We recruited 30 AD patients to undergo cue exposure through this VR cue exposure scene. The results showed that all AD patients reported that the scene was realistic, and the scene effectively induced the cue reactivity of AD patients.

### Participants

#### Sample size

The estimated sample size was calculated using the G*Power 3.1.9.7 sample size calculator. Sample size calculation was based on Wilcoxon-Mann-Whitney test (two groups), whereby the type I error was 0.05, the power was 0.8, the allocation ratio was 1:1, and the effect size was set as 0.84, which was cited from a randomized trial that compared the efficacy of virtual reality therapy and general treatment in alleviating alcohol craving in alcohol-dependent patients [41]. The estimated total sample size needed was 50, in which 25 subjects per group are required.

#### Recruit participants

From May 2022 to October 2022, 57 male patients with alcohol dependence who were hospitalized in the Fifth Department of Psychiatry, Second Affiliated Hospital of Xinxiang Medical University were recruited and divided into the study group (n = 29) and the control group (n = 28) according to a random number table.

Inclusion criteria: ① meet the diagnostic criteria for alcohol dependence in ICD-10; ② aged 18 ~ 50 years, Han nationality, primary school education or higher, right-handed; ③ acute withdrawal treatment ≥ 7 days, Clinical Alcohol Withdrawal Symptoms Rating Scale (CIWA-Ar) < 8; ④ normal vision or corrected vision; ⑤ normal hearing or corrected hearing; ⑥ no history of abuse of other psychoactive substances (except tobacco). Exclusion criteria: ① serious physical illnesses such as cardiovascular and cerebrovascular diseases; ② serious neurological illnesses such as epilepsy; ③ combined with other psychiatric illnesses; ④ unable to adapt to the virtual reality environment. Dropout criteria: ① subjects were unwilling to continue treatment and withdrew on their own; ② unable to complete treatment as prescribed for various reasons; ③ had missing data due to instrument failure.

The study protocol was reviewed and approved by the Ethics Committee of the Second Affiliated Hospital of Xinxiang Medical University, and all study subjects were fully informed of the purpose of the study and signed the informed consent form.

### Research technique

#### Measurement tools

A questionnaire was developed to collect general demographic information about the patient such as age, ethnicity, years of education, employment status, etc. as well as information on alcohol use including age of first drink, cumulative duration of use, duration of dependence, type of alcohol consumed, and dose of alcohol consumed etc.

Visual analogue scale (VAS) [42]: To assess the patient’s subjective psychological craving for alcohol. The score ranges from 0 to 10 points. “0” means that there is no subjective craving for alcohol, and “10” means that the subjective craving for alcohol is extremely strong and very difficult to control.

Physiological indicators were collected by multiparameter biofeedback (Infiniti3000A, Nanjing Vishee Medical Technology Co., Ltd.), including heart rate, skin conductance and respiratory signals before and during VR cue exposure.

#### Procedure

VR cue exposure procedure: The researcher first briefly introduced the experimental procedure to the patients, conducted a simple training for the patients, instructed the patients on the operation method of the VR handle, and instructed the patients to choose a comfortable position to sit on the chair. Alcohol was used to clean the patients’ skin. After the alcohol on the skin surface had evaporated, the BVP sensor was attached to the patients’ middle finger, the SC sensor was attached to the patients’ index finger and ring finger, and the respiratory sensor was attached to the patients’ chest or abdomen. After starting the procedure, the connection status was checked, and the patients were instructed to relax. If the connection status was good, heart rate, skin conductance and respiratory signals were collected for 2 min as baseline physiological index data, and then the patients were asked about alcohol craving by VAS as a baseline subjective assessment. The patients were then given a VR device and given enough time to adapt to the VR environment. After they had adapted, they began to watch the VR cue exposure video. At the same time, olfactory stimulation was provided by real alcohol. During the exposure procedure, a multiparameter biofeedback device was used to collect the patients’ heart rate, skin conductance and respiration, and then the patients’ alcohol craving was asked again by VAS.

**Fig. 1:**
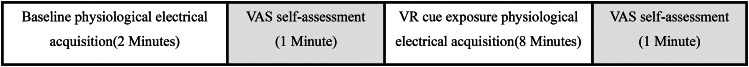
Flow chart of VR cue exposure

VR cue exposure was carried out in the hospital ward, and AD patients were permitted to stop the procedure at any time if they felt uncomfortable. After the conclusion of the VR cue exposure, participants were observed for about 10 min to ensure that they did not experience any significant discomfort. The sessions were conducted between 6:00 pm and 8:00 pm, as shown in Fig. 1.

**Fig. 2:**
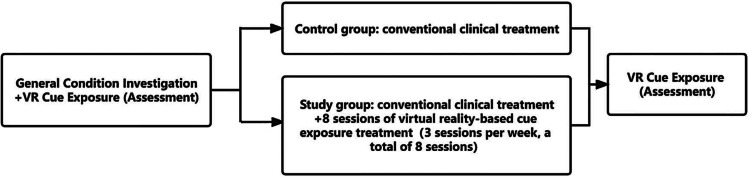
Flow chart of test procedure

Testing procedure: After completion of the acute withdrawal treatment, the alcohol-dependent patients were exposed to the above VR cue exposure (assessment), and the changes in VAS, heart rate, skin conductance, and respiration before and after exposure were recorded. Patients were randomly divided into the study group and the control group using the random number table method. The control group only received conventional clinical treatment for alcohol dependence, that is, benzodiazepines (such as oxazepam tablets) at the start of admission to control the acute withdrawal reaction, as well as high doses of group B and C vitamins, to correct water and electrolyte imbalances, and to improve liver function, and other symptomatic supportive treatments were given. The study group received conventional clinical treatment with the addition of VR cue exposure (treatment), 3 sessions per week (1 day apart) for 8 min. After treatment, a VR cue exposure (assessment) was conducted, and the changes in VAS, heart rate, skin conductance, and respiration before and after the exposure were recorded, as shown in Fig. 2.

### Statistical method

SPSS22.0 statistical software was used for data analysis, the Shapiro‒Wilk test was used for the normality test, and the measurement data conforming to the normal distribution were expressed as the mean ± standard deviation ($\mathop x\limits^ - \, \pm \,s$). The independent samples t test was used for comparison between groups, and the paired t test was used for comparison within groups. Measurement data not conforming to the normal distribution were presented as median and interquartile range [*M*(*P*25, *P*75)], the Mann‒Whitney U test was used for comparison between groups, and the paired Wilcoxon test was used for comparison within groups. *P* < 0.05 was considered statistically significant.

## Results

### General information

A total of 44 patients completed the study, 23 patients in the study group and 21 patients in the control group. 6 patients in the study group were dropped, 4 patients were automatically dropped during the study and 2 patients had missing data due to instrument failure. 7 patients in the control group were dropped, 4 patients were automatically dropped during the study and 3 patients had missing data due to instrument failure.

The mean age of the study group was 34.30 ± 5.23 years, the years of education was 9.00 (9.00, 12.00) years, the cumulative drinking time was 16.00 ± 5.74 years, the addiction time was 3.00 (2.00, 5.00) years, and the daily drinking amount was 210.00 (130.00, 260.00) g/day. The mean age of the control group was 35.90 ± 4.99 years, the years of education was 9.00 (9.00, 12.00) years, the cumulative drinking time was 16.24 ± 6.55 years, the addiction time was 3.00 (2.00, 5.50) years, and the daily drinking amount was 168.00 (104.00, 208.00) g/day. There were no significant differences in age, years of education, cumulative drinking time, addiction time or daily drinking amount between the two groups (*P* > 0.05).

### Comparison of VAS and physiological indicators before and after cue exposure before treatment

After VR cue exposure, the score of patients’ subjective psychological craving level 5.00 (2.00, 7.88) was significantly higher than that before exposure 2.00 (0.00, 3.75), the heart rate 87.67 ± 11.72 beats/min was significantly faster than that before exposure 83.64 ± 12.07 beats/min, the skin conductance 3.14 (1.42, 6.97) µΩ was significantly higher than that before exposure 2.68 (1.30, 6.02) µΩ, and the respiration 20.65 ± 2.84 times/min was significantly faster than that before exposure 20.04 ± 2.93 times/min (*P* < 0.05).

### Comparison of the changes in VAS and physiological indicators before and after cue exposure between the two groups before treatment

Before treatment, the changes in VAS, heart rate, skin conductance, and respiration before and after cue exposure were not significantly different (*P* > 0.05), as shown in Table 1.

**Table 1:** Comparison of the changes in VAS and physiological indicators before and after cue exposure between the two groups before treatment

		Study Group (n = 23)	Control group (n = 21)	*t/Z*	*P*
VAS (points)	Before exposure	3.00 (0, 5.00)	2.00 (0, 3.00)		
	After exposure	6.00 (2.00, 8.00)*	4.00 (2.00, 6.25)*		
	d (After-Before)	3.00 (2.00, 4.00)	2.00 (2.00, 3.25)	-0.827	0.408
Heart rate (beats/min)	Before exposure	83.41 ± 14.34	83.90 ± 9.32		
	After exposure	88.05 ± 13.01*	87.25 ± 10.43*		
	d (After-Before)	4.64 ± 3.54	3.36 ± 2.58	1.369	0.178
Skin conductance (µΩ)	Before exposure	2.70 (1.23, 6.17)	2.58 (1.46, 6.37)		
	After exposure	3.21 (1.40, 7.20)*	2.96 (1.46, 6.82)		
	d (After-Before)	0.18 (0, 0.47)	0.06 (-0.06, 0.34)	-1.070	0.285
Respiration (times/min)	Before exposure	19.66 ± 2.81	20.46 ± 3.06		
	After exposure	20.33 ± 2.75*	21.01 ± 2.96*		
	d (After-Before)	0.67 ± 1.33	0.55 ± 1.09	0.328	0.744

$\mathop x\limits^ - \, \pm \,s$Indicates the measurement data conforming to the normal distribution, M (P25, P75) indicates the measurement data not conforming to the normal distribution

*Indicates a significant change after exposure compared to before exposure (P < 0.05)

### Comparison of the changes in VAS and physiological indicators before and after cue exposure between the two groups after treatment

After treatment, the changes in VAS and heart rate in the study group before and after cue exposure were significantly lower than the control group (*P* < 0.05), while the changes in skin conductance and respiration between the study group and the control group were not statistically significant (*P* > 0.05), as shown in Table 2.

**Table 2:** Comparison of the changes in VAS and physiological indicators before and after cue exposure between the two groups after treatment

		Study Group (n = 23)	Control group (n = 21)	*t/Z*	*P*
VAS (points)	Before exposure	0 (0, 2.00)	2.00 (0, 3.00)		
	After exposure	2.00 (0, 4.00)*	4.00 (2.00, 5.50)*		
	d (After-Before)	1.00 (0, 2.00)	2.00 (2.00, 3.00)	-2.486	0.013
Heart rate (beats/min)	Before exposure	89.58 ± 13.89	86.25 ± 10.39		
	After exposure	91.15 ± 12.89*	89.34 ± 10.35*		
	d (After-Before)	1.57 ± 1.91	3.08 ± 2.78	-2.117	0.040
Skin conductance (µΩ)	Before exposure	1.52 (0.82, 2.32)	1.46 (0.80, 2.78)		
	After exposure	1.79 (1.02, 2.44)*	1.83 (1.20, 2.98)		
	d (After-Before)	0.10 (0, 0.25)	0.05 (-0.07, 0.25)	-0.717	0.473
Respiration (times/min)	Before exposure	20.25 ± 3.31	20.01 ± 2.22		
	After exposure	20.96 ± 3.37*	20.55 ± 2.09		
	d (After-Before)	0.71 ± 1.20	0.54 ± 1.02	0.452	0.653

$\mathop x\limits^ - \, \pm \,s$Indicates the measurement data conforming to the normal distribution, M (P25, P75) indicates the measurement data not conforming to the normal distribution

*Indicates a significant change after exposure compared to before exposure (P < 0.05)

### Comparison of the changes in VAS and physiological indicators before and after cue exposure in the study group before and after treatment

After treatment, the changes in VAS and heart rate in the study group before and after cue exposure were significantly lower than those before treatment (*P* < 0.05), while the changes in skin conductance and respiration were not significantly different from those before treatment (*P* > 0.05), as shown in Table 3.

**Table 3:** Comparison of the changes in VAS and physiological indicators before and after cue exposure in the study group before and after treatment

		Before treatment	After treatment	*t/Z*	*P*
VAS (points)	Before exposure	3.00(0, 5.00)	0 (0, 2.00)		
	After exposure	6.00 (2.00, 8.00)*	2.00 (0, 4.00)*		
	d (After-Before)	3.00 (2.00, 4.00)	1.00 (0, 2.00)	-3.304	0.001
Heart rate (beats/min)	Before exposure	83.41 ± 14.34	89.58 ± 13.89		
	After exposure	88.05 ± 13.01*	91.15 ± 12.89*		
	d (After-Before)	4.64 ± 3.54	1.57 ± 1.91	-4.380	< 0.001
Skin conductance (µΩ)	Before exposure	2.70 (1.23, 6.17)	1.52 (0.82, 2.32)		
	After exposure	3.21 (1.40, 7.20)*	1.79 (1.02, 2.44)*		
	d (After-Before)	0.18 (0, 0.47)	0.10 (0, 0.25)	-1.088	0.277
Respiration rate (times/min)	Before exposure	19.66 ± 2.81	20.25 ± 3.31		
	After exposure	20.33 ± 2.75*	20.96 ± 3.37*		
	d (After-Before)	0.67 ± 1.33	0.71 ± 1.20	0.084	0.934

$\mathop x\limits^ - \, \pm \,s$ Indicates the measurement data conforming to the normal distribution, M (P25, P75) indicates the measurement data not conforming to the normal distribution

*Indicates a significant change after exposure compared to before exposure (P < 0.05)

### Comparison of the changes in VAS and physiological indicators before and after cue exposure in the control group before and after treatment

After treatment, the changes in VAS, heart rate, skin conductance and respiration in the control group were not significantly different from those before treatment (*P* > 0.05). as shown in Table 4.

**Table 4:** Comparison of the changes in VAS and physiological indicators before and after cue exposure in the control group before and after treatment

		Before treatment	After treatment	*t/Z*	*P*
VAS (points)	Before exposure	2.00 (0, 3.00)	2.00 (0, 3.00)		
	After exposure	4.00 (2.00, 6.25)*	4.00 (2.00, 5.50)*		
	d (After-Before)	2.00 (2.00, 3.25)	2.00 (2.00, 3.00)	-0.574	0.566
Heart rate (beats/min)	Before exposure	83.90 ± 9.32	86.25 ± 10.39		
	After exposure	87.25 ± 10.43*	89.34 ± 10.35*		
	d (After-Before)	3.36 ± 2.58	3.08 ± 2.78	-0.277	0.784
Skin conductance (µΩ)	Before exposure	2.58 (1.46, 6.37)	1.46 (0.80, 2.78)		
	After exposure	2.96 (1.46, 6.82)	1.83 (1.20, 2.98)		
	d (After-Before)	0.06 (-0.06, 0.34)	0.05 (-0.07, 0.25)	-0.365	0.715
Respiration rate (times/min)	Before exposure	20.46 ± 3.06	20.01 ± 2.22		
	After exposure	21.01 ± 2.96*	20.55 ± 2.09		
	d (After-Before)	0.55 ± 1.09	0.54 ± 1.02	-0.025	0.980

$\mathop x\limits^ - \, \pm \,s$ Indicates the measurement data conforming to the normal distribution, M (P25, P75) indicates the measurement data not conforming to the normal distribution

*Indicates a significant change after exposure compared to before exposure (P < 0.05)

## Discussion

### Feasibility of the VR cue exposure scene

In this study, it was found that before treatment, the VAS score of patients after cue exposure was significantly higher than that before exposure, heart rate was significantly faster than that before exposure, skin conductance was significantly higher than that before exposure, and respiration was significantly faster than that before exposure. The results are consistent with previous studies (Bordnick et al. [9], Ghita et al. [11], Wang W et al. [13], Reid et al. [15], Jansma et al. [16]), suggesting that VR cue exposure can effectively induce psychological craving and physiological responses in AD patients.

### Effects of virtual reality-based cue exposure therapy

Currently, studies in the field of substance use disorders have mainly evaluated the effectiveness of CET as follows [31]. First, the changes in subjective craving levels and physiological responses before and after cue exposure were assessed before treatment. Second, the changes in subjective craving levels and physiological responses before and after cue exposure were assessed after treatment. Third, the overall treatment effect was determined by comparing the changes in subjective craving levels and physiological responses before and after treatment. In this study, there was no significant difference in the change in VAS before and after cue exposure between the two groups before treatment (*P* > 0.05), indicating that the two groups were at the same baseline level. After treatment, the change in VAS before and after cue exposure in the study group was significantly lower than that in the control group, and the change in VAS in the study group during exposure to the alcohol cue was significantly lower than that before treatment, while there was no significant change in the control group compared with that before treatment. It is showed that VR-CET can effectively reduce subjective craving in AD patients, which is consistent with previous studies (Lee JH et al. [43], Lee SH et al. [41]). However, in this study, AD patients remained highly alcohol craving when re-exposed to alcohol-related cues after treatment in both the control and study groups. On the one hand, this may be due to the short research period of this study, and the complete elimination of psychological craving is a long process; on the other hand, it may be due to the short intervention period and fewer interventions in this study, and the duration and intensity of the intervention may be increased in the future to achieve better treatment outcomes.

In this study, there were no significant differences in the changes in heart rate, skin conductance, and respiration before and after cue exposure before treatment (*P* > 0.05), indicating that the two groups were at the same baseline level. After treatment, the change in heart rate before and after cue exposure in the study group was significantly lower than that in the control group, and the study group was significantly lower than before treatment, while there was no significant change in the control group compared with before treatment. It is showed that VR-CET can effectively reduce the magnitude of heart rate variability during cue exposure in AD patients. However, after treatment, there were no significant differences in the changes in skin conductance and respiration between the study group and the control group before and after cue exposure (*P* < 0.05). There were no significant changes in the changes in skin conductance and respiration before and after cue exposure in the study and control groups compared with that before treatment, and the difference was not statistically significant (*P* < 0.05). This study suggests that heart rate may be a sensitive objective indicator of craving, and the result is consistent with Ooteman et al. [18]. Breathing is a physiological indicator that can be consciously controlled, and we found that some alcohol-dependent patients in this study consciously controlled the frequency and amplitude of their breathing when exposed to the cues. Although skin conductance is not controlled by consciousness, it is more affected by the external environment, such as environmental temperature and humidity, which may affect skin conductance. Therefore, future studies should consider and strictly control their influencing factors when monitoring physiological indicators.

### Deficiencies and prospects

However, there are some deficiencies in this study. First, the sample size of this study is small, and the study subjects are all male AD patients, resulting in the effect of VR cues exposed to induce and treat alcohol craving being gender different and not universal. Second, this study did not design a traditional CET as a control group, and it is impossible to compare whether the VR-CET is better than the traditional CET. Finally, the intervention duration and intervention period of this study were short, and long-term follow-up was not conducted to understand the long-term effectiveness of VR-CET.

Future studies are still needed to further verify the relationship between heart rate, skin conductance, and respiration and craving. Future studies could also add aversion therapy or relaxation therapy after CET to increase the efficacy of the treatment. In addition, it is possible to monitor the patient’s blood pressure changes and use eye-tracking technology to track the participants’ gaze objects, gaze duration and other information to explore objective physiological indicators that can effectively reflect craving and help in the assessment and treatment of alcohol-dependent patients.

### Conclusion

In conclusion, VR-CET can reduce the psychological craving and part of the physiological responses of AD patients during cue exposure in the short term, and may help in the treatment of AD.

## Data Availability

The datasets generated and analysed during the current study are not publicly available due to patient privacy regulations but are available from the corresponding authors on reasonable request.
